# Slope stability considering multi-fissure seepage under rainfall conditions

**DOI:** 10.1038/s41598-024-62387-3

**Published:** 2024-05-22

**Authors:** Jianqing Jia, Chengxin Mao, Victor O. Tenorio

**Affiliations:** 1 School of Traffic and Transportation, Lanzhou Jiaotong University, Lanzhou, 730070 China; 2 Department of Mining and Geological Engineering, University of Arizona, Tucson, AZ 85721 USA

**Keywords:** Seepage, Multi-fissure, Strength reduction, Rainfall infiltration, Civil engineering, Natural hazards

## Abstract

Fissures form the channel for rainwater infiltration, which accelerate the infiltration of rainwater into slope bodies, hence its important impact on the seepage field and stability of the slope. In this paper, taking one landslide of Liang-Wan freeway as the research object, firstly, the equivalent permeability coefficient method is used to homogenize the fissured soil. Then considering the boundary conditions of rainfall infiltration and groundwater level, a fluid–structure coupling model is established based on saturated–unsaturated seepage theory, and evolution characteristics of seepage, displacement and stress of the slope are studied. Based on these, the slope stability coefficient is determined. The results show that the rising rate of pore water pressure and volume water content of topsoil increases when multi-fissure seepage is considered, and the pore water velocity is larger in the local seepage range of fissures. With the increase of buried depth, the closer to groundwater level, the influence of multi-fissure seepage gradually weakens. The theoretical calculation results of slope displacement are more consistent with the field monitoring results. With the increase of rainfall time, the stability coefficient of slope decreases gradually, and the rate and range of decrease are greater.

## Introduction

The existence of fissures will affect the physical and mechanical properties of soil and rock mass, such as permeability, strength, deformation characteristics, etc. and when rainfall, rainwater will preferentially penetrate into the slope through the seepage channels to form seepage, which will seriously affect slope stability^1–4^. Therefore, this issue is one of the long-term hot topics in the field of slope engineering. Zeng et al.^5^ studied rainfall infiltration affected by fissure number, angle and depth, and the permeability coefficient ratio. Based on these, slope stability is also studied thinking about the effect of fissure seepage anisotropy on slope stability is discussed using the unsaturated seepage theory. Cao et al.^6^ studied the fissure evolution, rainfall infiltration and scouring using model test, analyzed the effect of fissure development and rainfall to soil seepage of red clay slope. Zhou et al.^7^ put forward a two-domain infiltration model of dominant flow in fissured clay considering fissure area ratio, explored the effects of rainfall intensity, fissure area ratio and saturated permeability coefficient of fissure domain on water accumulation time, infiltration amount and infiltration depth in soil matrix domain and fissure domain, and revealed the infiltration law after dry shrinkage fissuring of soil. Shi et al.^8^ used the seepage stress coupling model of anisotropic rock mass based on equivalent continuum model and Louis empirical formula to simulate and analyze the anisotropic seepage law of layered fissure-slope along the dip. Zeng^9^ used Morgenstern-Price method considering matrix suction to analyze the stability of loess slopes with different rainfall intensities and different rainfall durations and thought that the saturated area in the deep layer of soil or upper stagnant water formed by rainfall under the action of seepage channel was the main factor of slope instability. Based on the limit equilibrium theory, Fu et al.^10^ derived the expression of anti-sliding stability coefficient of rock slope considering rock mass damage and groundwater seepage and calculated the stability factor of rock slope under various conditions. Ni et al.^11^ analyzed the formation mechanism of the hydraulic path of seepage in fissured rock mass and compiled the analysis program, and then studied the main forms of the hydraulic path and its stress correlation. Wang et al.^12^ put forward the layout of slope fissures and studied the seepage characteristics and transient stability of soil slopes with fissures under rainstorm. Yuan et al.^13^ analyzed the changing rules of rainfall infiltration which is affected by the direction, degree, distribution of the soil anisotropy. Nian et al.^14^ used one dual-continuum model to analyze slope stability considering the influence of rainfall infiltration. Based on these, established a two-dimensional slope model to analyze the infiltration boundary under four types of rainfall intensity.

However, in the present study of the influence of multi-fissure seepage on slope stability, the changing laws of seepage field is mainly taken as the research object, and the influence of multi-fissure on seepage field^15–17^ and slope stability under rainfall condition is not considered at the same time. Therefore, taking one soil slope as an example, a fluid–structure coupling model is established considering multi-fissure seepage, the stress, plastic strain, displacement, pore pressure and flow velocity are all calculated, and the stability factor of the slope under different rainfall conditions are also determined.

## Project overview

### Engineering background

The landslide project of Liangwan freeway is located at Fenshui Town, Wanzhou District, China. The leading edge of landslide is located in the gentle slope area at the top of retaining wall on the inner side of freeway, and the trailing edge is located at the foot of rocky steep cliff. The left and right boundaries are distributed along the micro-ridge line from top to bottom. The occurrence of rock strata is 145–170°∠18–21°, and no fault is found in this area. There are two types of groundwater in landslide area: pore water of loose rock and fissure water of bedrock. The project area belongs to subtropical warm and humid monsoon climate zone, with an average annual rainfall of about 1052.7 mm.

### Landslide monitoring

**Figure 1:**
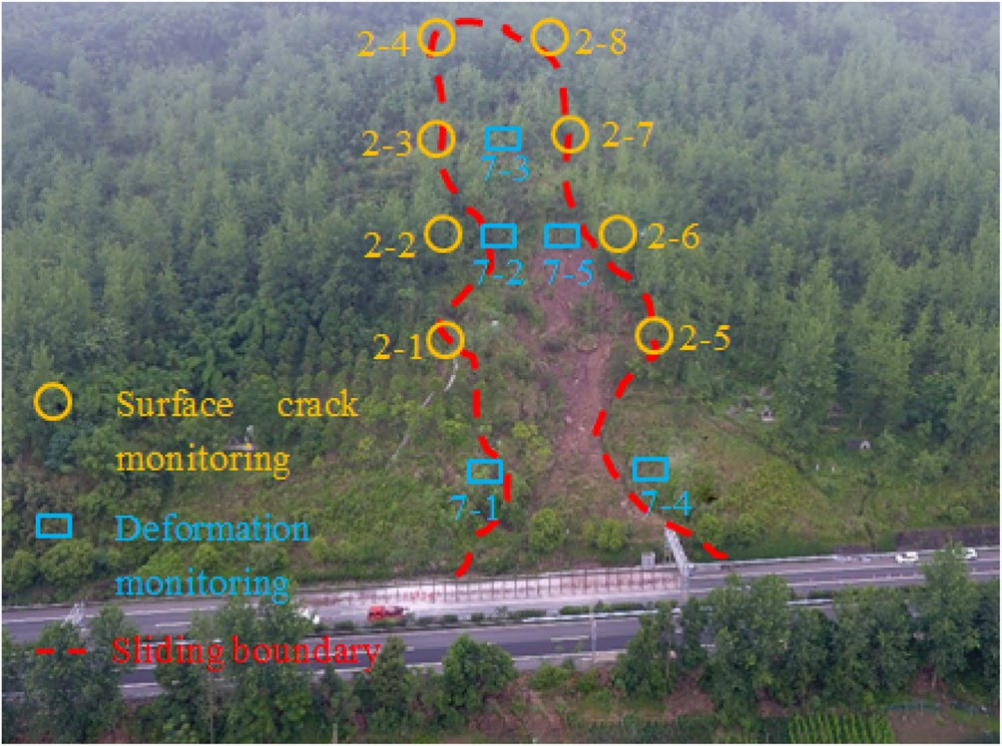
Monitoring points.

**Figure 2:**
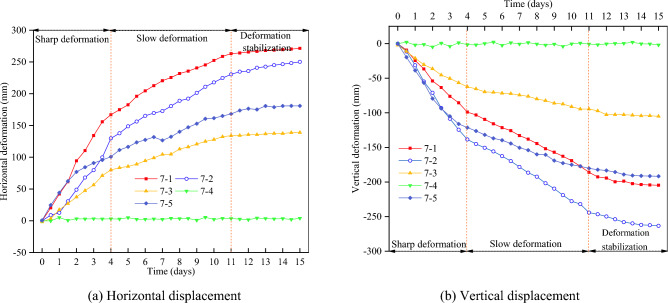
Deformations of monitoring points.

The section K1515 + 860 ~ K1516 + 000 in the downward direction of Liangwan freeway is affected by continuous rainfall, and the excavated slope on the right side is deformed and slipped. According to the field investigation, the slope has a direction of 345° and an angle of nearly 30°, and there is obvious deformation on the surface. The middle and front part of the slope partially collapses and slides, and longitudinal shear fissures can be seen within the deformation range. The extension length of fissures at the trailing edge of deformable bodies is about 60 m, and the extension length of fissures near the freeway side is about 120 m. The landslide height is about 75 m, with a length of about 180 m. The maximum tensile fissures of the slope are about 80 cm, and the visible depth is about 90 cm. In order to ensure the stability of slope engineering and accurately determine the sliding range and sliding surface^18,19^, the deformation monitoring points and surface cracks monitoring points are arranged on site to monitor and analyze the slope stability (as shown in Fig. 1), and the deformation of monitoring points are shown in Fig. 2.

As can be seen from Fig. 2, with the increase of monitoring time, the displacement value of each monitoring point mainly shows the following change process: (1) Sharp deformation. It lasts about 4 days, the average horizontal displacement value of each measuring point accounts for about 55% of its final horizontal displacement, and the average vertical displacement value of each measuring point accounts for about 52% of its final vertical displacement. (2) Slow deformation. It lasts about 7 days, the average horizontal displacement of each measuring point accounted for about 41% of its final horizontal displacement, and the average vertical displacement of each measuring point accounted for about 44% of its final vertical displacement. (3) Deformation stabilization. After 12 days of monitoring, the displacement change of each measuring point is very small, and the change rate tends to zero basically, which indicates that the horizontal displacement has tended to be stable. Because the monitoring points 7–1 and 7–2 are located on the main sliding surface of the sliding body, the displacement is large. The final horizontal displacement convergence values of 7–1 and 7–2 are 271.3 mm and 250.1 mm respectively, and the final vertical displacement convergence values are 204.6 mm and 263.3 mm respectively. Although 7–5 is not located on the main sliding surface of the sliding body, it is very close to the top of the slope and has a high terrain. When the slope landslide occurs, its vertical displacement is also large.

## Slope stability analysis considering multi-fissure seepage

### Computational theory

The governing equation of saturated–unsaturated seepage flow is^20–22^:

1 $$\frac{\partial }{\partial x}\left( {K_{x} \frac{\partial h}{{\partial x}}} \right) + \frac{\partial }{\partial y}\left( {K_{y} \frac{\partial h}{{\partial y}}} \right) = m_{w} \rho_{w} g\frac{\partial h}{{\partial t}}$$

where: the *K*_*x*_ and *K*_*y*_ is the saturated permeability coefficient in x and y directions respectively, h is the head of water, *m*_*w*_ is the specific water capacity, *ρ*_*w*_ is the density of water, g is the acceleration of gravity, t is the calculation time.

The governing equation of fluid–structure coupling is^23–26^:

2 $$\left\{ \begin{gathered} K + \{ H\} + \{ f\} = 0 \hfill \\ \{ \sigma \} = D\{ \varepsilon \} = DB\{ \delta \} \hfill \\ M\{ \delta \} = X + F \hfill \\ \end{gathered} \right.$$

where: *K* is the correlation matrix of permeability coefficient, $\{ f\}$ is the head distribution function of seepage field, $\{ H\}$ is a shape function, *F* is the permeability matrix, *M* is the whole stiffness matrix, *X* is the external load matrix of joints, *B* is the stress interpolation matrix, *D* is the elastic matrix.

### Numerical calculation model

Assuming that the permeability of soil in fissure area is much higher than that of undisturbed soil, the slope soil is divided into undisturbed soil and fissure soil, and the equivalent permeability coefficient method is used to homogenize the fissure soil^27,28^.

**Figure 3:**
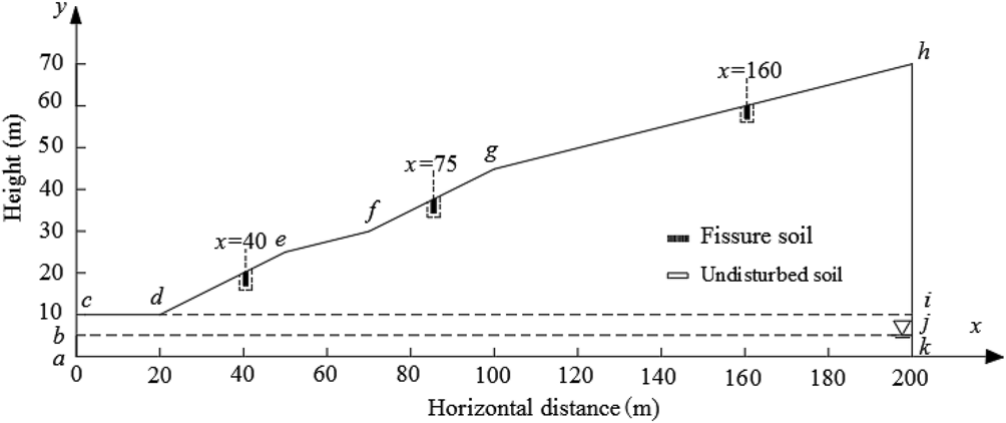
Calculation model.

In order to study the influence of multi-fissure seepage on slope stability under rainfall condition, a numerical analysis model is established, and the size of the model is shown in Fig. 3. According to the field monitoring results, the cumulative deformation of fissures 2–1, 2–2 and 2–3 is large, and they are located on the main sliding surface of the sliding body, so the fissures are set at the positions x = 40, x = 75 and x = 160 in the slope model respectively. In Fig. 3, bj is the groundwater level line, cdefgh is the rainfall infiltration boundary, and ac, cd and ik are the free drainage boundaries.

**Figure 4:**
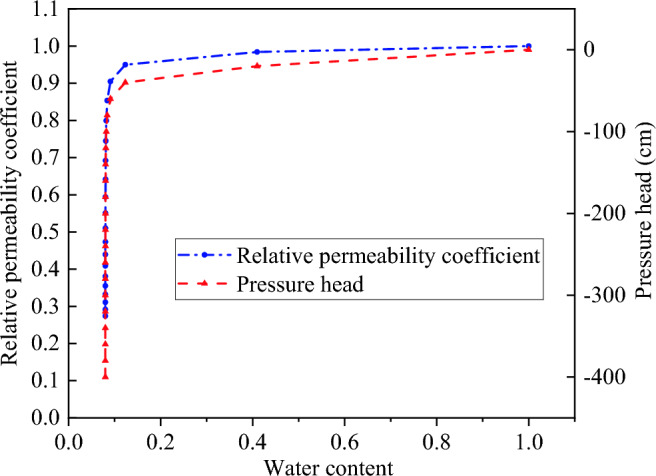
Characteristic curves of water and soil.

**Figure 5:**
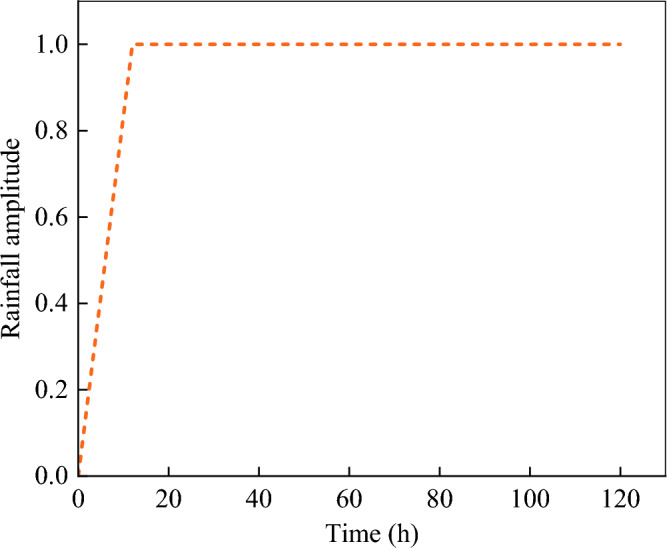
Rainfall amplitude curve.

The water–soil characteristic curve and permeability coefficient function curve are calculated by Van Genuchten model^23–25^, and they are shown as Fig. 4. The saturated permeability coefficient of undisturbed soil is 0.018 m/h, and the saturated permeability coefficient of fissured soil is 2–4 orders of magnitude higher than that of undisturbed soil^29,30^. In this paper, the saturated permeability coefficient of fissured soil is taken as 1.8 m/h. Based on the meteorological data of slope location, the rainfall intensity is 0.022 m/h, the rainfall duration is 120 h, and the rainfall amplitude curve is shown in Fig. 5. The initial void ratio is 1.

According to the field exploration, the parameters of sliding zone soil are shown in Table 1. The influence of soil saturation state change on elastic modulus and Poisson's ratio is not considered.

**Table 1:** Soil parameters.

Name	Saturation state	Weight (KN/m^−3^)	Cohesion (kPa)	Internal friction angle (°)	Elastic modulus (kPa)	Poisson's ratio
Undisturbed soil	Natural	19.8	28.7	14.6	40,000	0.3
	Saturated	20.5	22.7	12.8		
Fissured soil	Natural	12.6	8	15	35,000	0.32
	Saturated	13.9	5	12		

In order to consider the parameter change of undisturbed soil after saturation, the field variable reduction coefficient (FV1) and field variable saturation state (FV2) are controlled^29,30^, and the strength reduction obtained is shown in Table 2.

**Table 2:** Strength reduction.

*c*	*φ*	Reduction factor	*c'*	*φ'*	Saturation state	*c*	*φ*	Reduction factor	*c'*	*φ'*	Saturation state
28.7	14.6	0.5	57.4	27.51776	0	22.7	12.8	0.5	45.4	24.43652	1
28.7	14.6	0.75	38.26667	19.15249	0	22.7	12.8	0.75	30.26667	16.85292	1
28.7	14.6	1	28.7	14.6	0	22.7	12.8	1	22.7	12.8	1
28.7	14.6	1.25	22.96	11.77109	0	22.7	12.8	1.25	18.16	10.30137	1
28.7	14.6	1.5	19.13333	9.851381	0	22.7	12.8	1.5	15.13333	8.612724	1
28.7	14.6	1.75	16.4	8.46609	0	22.7	12.8	1.75	12.97143	7.397074	1
28.7	14.6	2	14.35	7.420448	0	22.7	12.8	2	11.35	6.480859	1
28.7	14.6	2.25	12.75556	6.603684	0	22.7	12.8	2.25	10.08889	5.765915	1
28.7	14.6	2.5	11.48	5.94831	0	22.7	12.8	2.5	9.08	5.192649	1
28.7	14.6	2.75	10.43636	5.410923	0	22.7	12.8	2.75	8.254545	4.722831	1
28.7	14.6	3	9.566667	4.962366	0	22.7	12.8	3	7.566667	4.330827	1

### Slope stability analysis considering multi-fissure seepage

**Figure 6:**
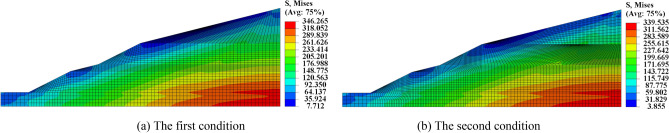
Mises stress nephogram.

**Figure 7:**
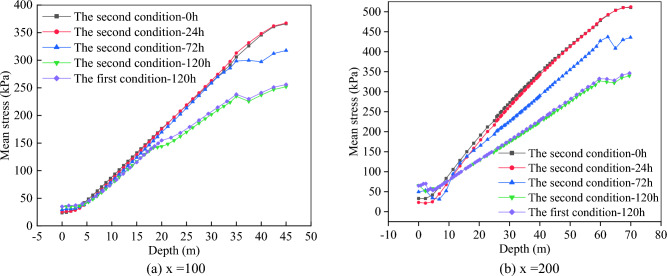
Mises stress variation curve.

We determine two kinds of condition. The first condition is not to consider multi-fissure seepage and the second condition is to consider multi-fissure seepage. After 120 h of rainfall, the Mises stress variation characteristics of slope of the first conditions and the second condition are shown in Fig. 6, and the Mises stress variation curves at the middle of slope (x = 100) and the top of slope (x = 200) are shown in Fig. 7.

It can be seen from Fig. 6 that the maximum Mises stress of the slope under the first condition is about 346.3 kPa. Because the slope under the second condition contains many fissures, these fissures form a channel for rainwater infiltration, which accelerates the infiltration of rainwater to the surface layer of the slope, and makes the Mises stress of the surface layer under the second condition greater. However, with the increase of rainfall duration, greater displacement will occur in the second condition, and the maximum Mises stress of slope is 339.5 kPa.

**Figure 8:**
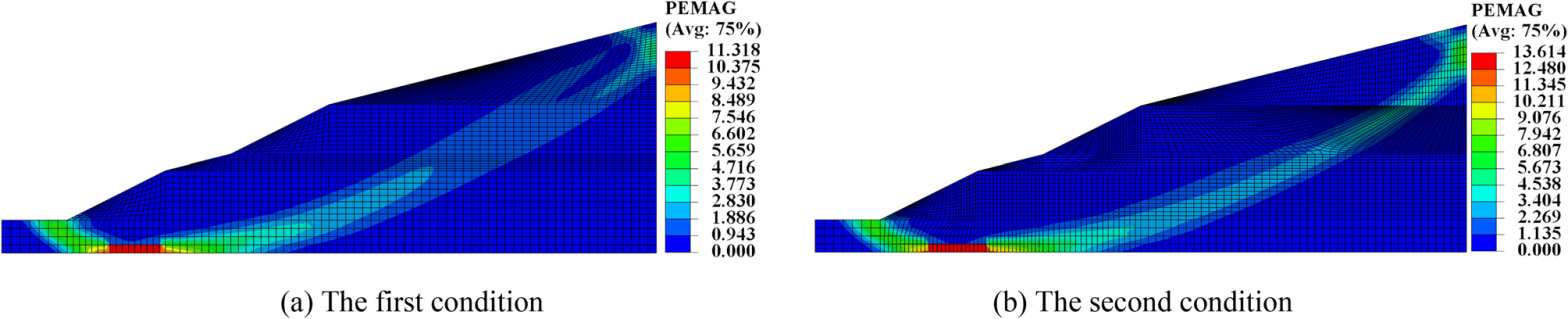
Plastic strain nephogram.

From Fig. 7, we can find that the Mises stress increases with the increase of buried depth owing to the stress is mainly the dead weight of soil. There is a downward trend in the depth range of 35–40 m in Fig. 7a and in the depth range of 0–10 m and 60–70 m in Fig. 7b, which indicates that the soil mass has obviously shifted in this depth range during rainfall. In the second condition, the Mises stress curves of rainfall 24 h and no rainfall basically coincide and the slope also has no displacement after rainfall 24 h, which show that the influence of rainwater gravity on stress is small. However, after 120 h of rainfall, the stress of the second condition is greater than that of the first condition at the same position of slope. The plastic strain of slope under the first condition and the second condition after 120 h of rainfall is shown in Fig. 8.

It can be seen from Fig. 8 that average width of the sliding-zone of the slope in the first condition is 11.3 m, and that in second condition is 13.6 m. At the same time, we find that the simulated sliding surface in second condition is closer to the potential sliding surface of the project.

**Figure 9:**
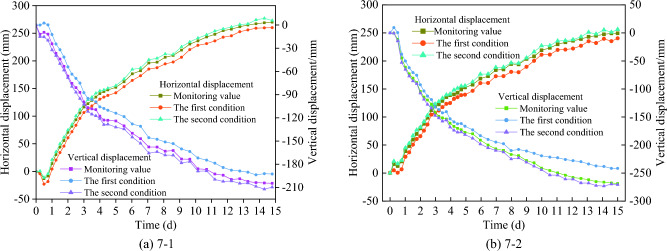
Displacement comparison curves.

In order to further determine the correlation between the two conditions and engineering practice, the field monitoring displacements are compared with the numerical simulation results of the first and second conditions, as shown in Fig. 9.

It can be seen from Fig. 9 that the variation curve of slope displacement in the second condition is very close to the field monitoring results, and the coincidence degree is higher. The maximum difference of vertical displacement is only 8.1 mm, and the maximum difference of horizontal displacement is only 6.8 mm.

**Figure 10:**
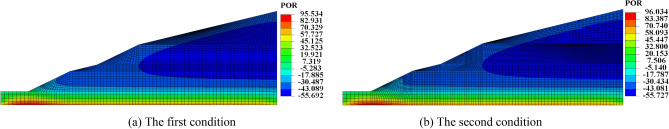
Soil pore pressure.

**Figure 11:**
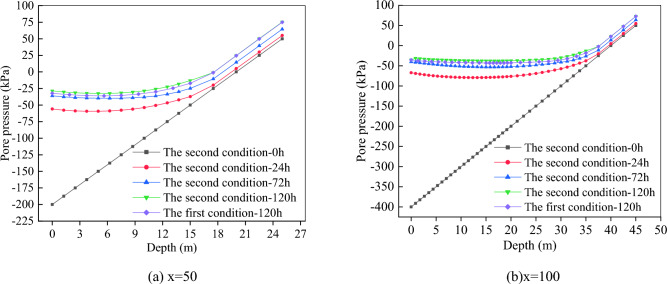
Soil pore pressure changing with buried depth.

After 120 h of rainfall, the pore pressure distribution of slope soil of the two conditions are shown in Fig. 10, and the variation curves of pore pressure along buried depth at the foot of slope (x = 50) and the middle of slope (x = 100) are shown in Fig. 11.

It can be seen from Fig. 10 that the distribution of soil pore water pressure is mainly affected by groundwater level and rainfall. After rainfall for 120 h, it is easier to saturate near the foot of the slope, while the top of the slope is relatively dry. The maximum soil pore pressures of the first and second conditions are 96.0 kPa and 95.5 kPa, respectively.

It can be seen from Fig. 11 that no matter at the position of x = 50 or x = 100, when there is no rainfall, the pore pressure is mainly affected by groundwater level, and changes linearly with depth. After rainfalls, the longer the rainfall lasts, the greater the pore pressure at the same buried depth. The pore pressure along the buried depth is to decrease first and then increase, and the decreasing range decreases with the increase of rainfall duration. Figure 11a shows that after 120 h of rainfall, the pore pressure in the range of 0–18 m is higher considering multi-fissure seepage than that of no fissure. It is mainly affected by groundwater level in the range of 18–25 m, and the curves basically coincide. Figure 11a shows that after 120 h of rainfall, the pore pressure in the range of 0–35 m is higher considering multi-fissure seepage than that of no fissure. It is mainly affected by groundwater level in the range of 35–45 m, and the curves basically coincide.

**Figure 12:**
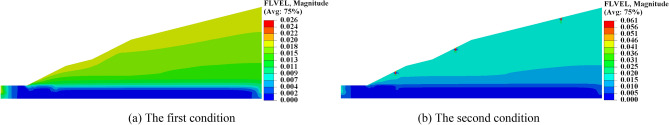
Flow velocity nephogram.

At the same time, we got the pore water velocity of the first and second conditions after rainfall for 120 h, which is shown in Fig. 12.

It can be seen from Fig. 12 that after 120 h of rainfall, the water will quickly enter the fissure and its velocity in the fissure is relatively large owing to high permeability of the fissure, which will form a small-scale seepage in the fissure area. When rainfall lasts longer, the slope soil will be saturated and the water velocity tends to be stable, but it is still affected by fissures. Figure 12 also shows that the pore-water velocity is relatively small in first condition. Water permeates mainly through pore and soil permeability is far less than that of fissures. With increase of soil moisture content, the water velocity also continues to increase, which is relatively slow compared with the second condition.

**Figure 13:**
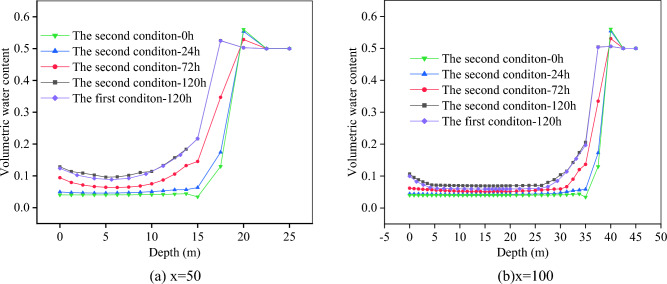
Soil volume moisture content.

The change characteristics of soil volume moisture content at the foot of slope (x = 50) and the middle of slope (x = 100) after 120 h of rainfall are shown in Fig. 13.

It can be seen from Fig. 13 that within 120 h, the longer the rainfall time, the higher the volume moisture content of slope surface. When the rainfall time is constant, the soil volume moisture content first decreases and then rises along the slope depth. It will be stable when the depth is 22.5 m (shown in Fig. 13a), and it will be stable when the depth is 42.5 m (shown in Fig. 13b). After 120 h of rainfall, when the depth range is 0–11.3 m (x = 50), the volume water content of the second condition is higher due to the influence of fissure seepage. When the depth ranges from 11.3 to 25 m (x = 50), the influence of seepage is weakened, and the volume water content of the first and second conditions is mainly affected by groundwater, and their curves almost coincide. In the depth range of 0–32.2 m (x = 100), the volume moisture content of the second condition is higher. When the depth ranges from 32.2 to 45 m (x = 100), the volumetric moisture contents of the first and second conditions are almost equivalent.

**Figure 14:**
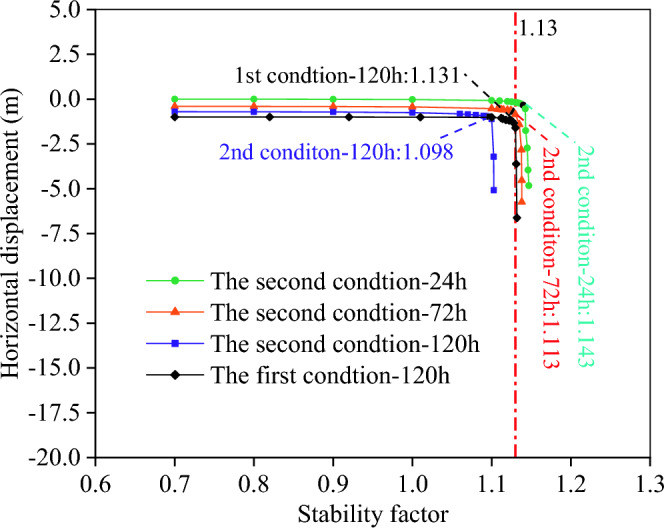
Stability coefficient of the slope.

In this paper, the inflection point of characteristic point displacement is taken as the evaluation standard of stability coefficient^18^, and the change characteristics of slope stability coefficient at different times after rainfall are shown in Fig. 14. The stability cofficient of different calculation method and condtions are shown in Table 3.

**Table 3:** Stability coefficient of different calculation method and condtions.

Methods	Working conditions			
	Natural conditions	Rainfall conditions		
		24 h	72 h	120 h
Limit equilibrium method	1.20	1.13		
The first condition	1.195	1.165	1.152	1.131
The second condition	1.192	1.143	1.113	1.098

As can be seen from Fig. 14 and Table 3, with rainfall time increase, the stability coefficient of slope gradually decreases. The decreasing rate of the second condition is higher than that of the first condition, which shows that multi-fissure seepage has great influence on slope stability. After 120 h of rainfall, the slope stability coefficient of the second condition is 1.098, which is 3.0% lower than that of the first condition and 2.9% lower than that calculated by limit equilibrium method, indicating that the slope stability state determined by limit equilibrium method or without considering multi-fissure seepage is dangerous.

## Conclusions

In this paper, the influences of groundwater and soil saturation state to slope stability are considered, the different condition of multi-fissured slope are determined. Based on these, the items including plastic strain, slope displacement, pore-pressure, flow velocity and soil volume moisture content etc. are studied. Finally, the stability coefficient of the slope is calculated and analyzed by strength reduction method, and compared with non-fissured condition and the monitoring results of the project site. The following main conclusions are drawn:

1. When considering multi-fissure seepage, fissure forms channel for rainwater seepage, which accelerates the seepage of rainwater to the surface layer of slope makes the rising rate of pore water pressure and volume moisture content of slope surface layer increase, and the pore water velocity is larger. With increase of buried depth, the closer to groundwater level, the influence of multi-fissure seepage gradually weakens.
2. When considering the multi-fissure seepage in study of slope stability, the calculated slope slip-surface is closer to the actual slip-surface of the project, and the displacement curve is closer to the field monitoring results. All of these show that the calculated results of slope stability coefficient are more accurate considering multi-fissure seepage.
3. With increase of rainfall time, the stability coefficient of slope decreases gradually, and the decreasing rate and decreasing range are greater. It can be seen that multi-fissure seepage has great influence on slope stability.

## Data Availability

All data, models, and code generated or used during the study appear in the submitted article.
